# Supplementary material for: Unveiling the Structural Characteristics and Bioactivities of the Polysaccharides Extracted from Endophytic Penicillium sp
Source: Molecules. 2023 Jul 31;28(15):5788. doi: 10.3390/molecules28155788 (PMC10421393; doi:10.3390/molecules28155788)
Supplement: Supplementary file 1 [file molecules-28-05788-s001.zip › molecules-2506648-supplementary.pdf]

Supplementary materials

Unveiling the structural characteristics and bioactivities of the polysaccharides extracted from endophytic *Penicillium* sp.

Kumar Vishven Naveen[1], Anbazhagan Sathiyaseelan[1], Sumana Mandal[2], Kiseok Han[1], and Myeong-Hyeon Wang[1]*

[1]Department of Bio-Health Convergence, Kangwon National University, Chuncheon 200-701, Republic of Korea

[2]Department of Chemistry, Sungkyunkwan University, Suwon 16419, Republic of Korea

***Corresponding author**

Myeong-Hyeon Wang

Email: mhwang@kangwon.ac.kr

Chemicals and consumables

D-(+)-glucose (dextrose) powder was obtained from Yakuri pure chemicals Co., Ltd., Japan. Potato dextrose agar (PDA; Difco^{TM}) were obtained from Becton, Dickinson and Co., USA. DEAE Sepharose fast flow was acquired from GE Healthcare, Sweden. The standard monosaccharides (L-arabinose, D-glucose, D-galactose, D-mannose, L-rhamnose, and D-xylose), peptone, Folin-Ciocalteu's phenol reagent, potassium acetate, 2,2-diphenyl-1-picrylhydrazyl (DPPH), 2,2′-azino-bis(3-ethylbenzothiazoline-6-sulfonic acid) diammonium salt (ABTS), propidium iodide (PI), acridine orange (AO), ethidium bromide (EB), and rhodamine-123 (Rh-123) were supplied by Sigma Aldrich, Republic of Korea (ROK). Ethyl alcohol (EtOH), methyl alcohol (MeOH), L-(+)-ascorbic acid (AA) and n-butyl alcohol were purchased from Daejung Co., Ltd., ROK. Chloroform was purchased from Samchun pure chemicals Co., Ltd., ROK. Dulbecco's Modified Eagle Medium (DMEM) and Roswell Park Memorial Institute (RPMI) medium were acquired from ThermoFisher Scientific, ROK. Phosphate Buffered Saline (PBS) was obtained from Corning®, USA. Fetal bovine serum (FBS), penicillin-streptomycin (PS) solution were purchased from Hyclone Laboratories, USA. CELLOMAX^{TM} viability assay kit (WST) was provided by MediFeb, ROK. The apoptosis assay kit (Annexin V FITC; and PI) was supplied by ThermoFisher Scientific, USA. The cell line such as the human embryonic kidney 293 (HEK-293), mouse embryonic fibroblast (NIH3T3), and human prostate cancer (PC-3) cells were procured from the Korean Cell Line Bank (KCLB), ROK.

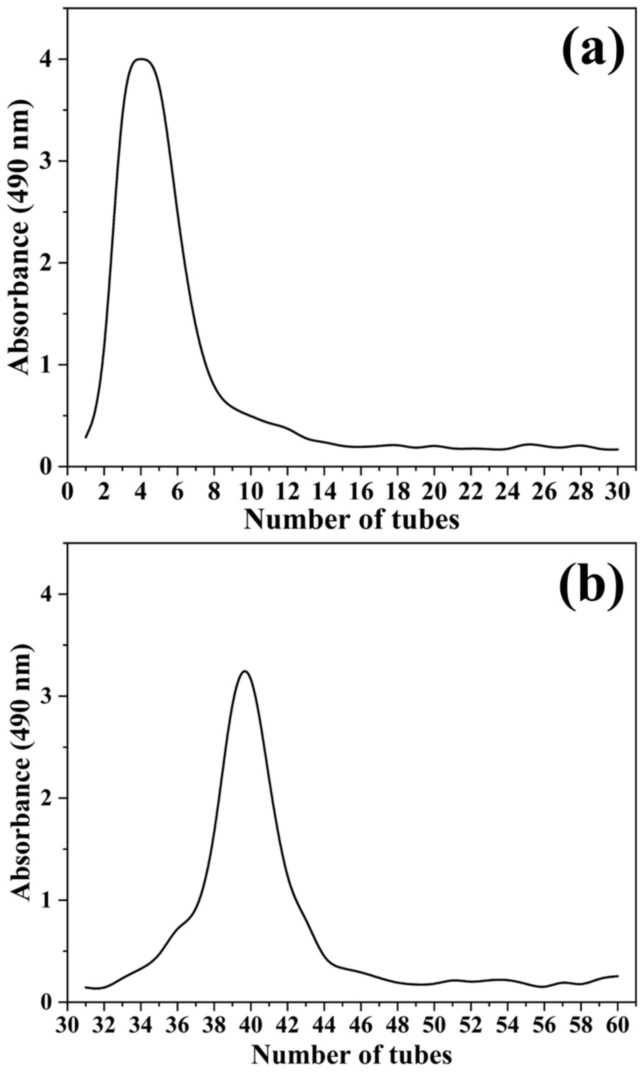

Figure S1. The elution curve of IPS-1 (**a**) and IPS-2 (**b**) eluted with deionized water and 0.1 mol/L of NaCl, respectively on DEAE-Sepharose column and examined using phenol-sulfuric acid assay.

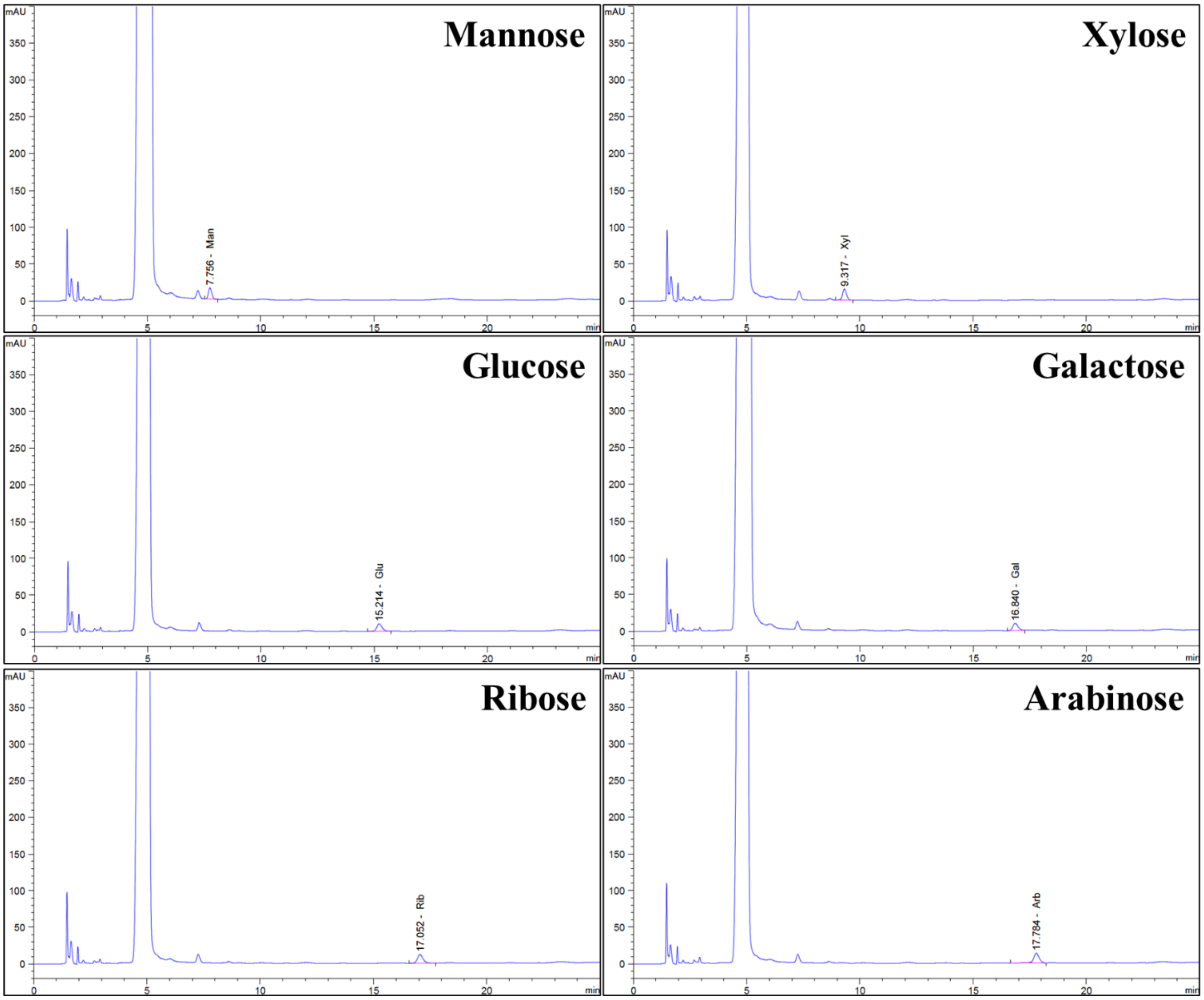

Figure S2. Chromatogram of the standard monosaccharides determined using high-performance liquid-chromatography (HPLC) coupled with UV detector.

Table S1. Analysis of the *in-vitro* antioxidant activities and cytotoxic activities of intracellular polysaccharides (IPS-1 and IPS-2) extracted from endophytic *Penicillium radiatolobatum* compared to standard ascorbic acid (AA).

Sample	Half maximal inhibitory (IC_{50}) concentration (μg/mL)					
	ABTS radical scavenging	DPPH radical scavenging	Ferric reducing power	Cytotoxic activity		
				HEK-293 cells	NIH3T3 cells	PC-3 cells
IPS-1	223 ± 2.0	643 ± 2.5	>1000	>1000	>1000	>1000
IPS-2	108 ± 2.5	272 ± 4.0	760 ± 5.0	>1000	>1000	435 ± 3.0
AA	54.5 ± 1.25	172 ± 2.5	405 ± 3.5	-	-	-
